# Sleep Deprivation Does Not Influence Photic Resetting of Circadian Activity Rhythms in Drosophila

**DOI:** 10.3390/clockssleep4010018

**Published:** 2022-03-21

**Authors:** David C. Negelspach, Sevag Kaladchibachi, Hannah K. Dollish, Fabian-Xosé Fernandez

**Affiliations:** 1 Department of Psychology, University of Arizona, Tucson, AZ 85721, USA; davidnegelspach@arizona.edu (D.C.N.); sevag.kaladchibachi@mail.utoronto.ca (S.K.); 2 Graduate Interdisciplinary Program in Neuroscience, University of Arizona, Tucson, AZ 85721, USA; hannahdollish@email.arizona.edu

**Keywords:** light, circadian, sleep deprivation, insomnia, phase shift, drosophila

## Abstract

Previous investigations in humans and rodent animal models have assessed the interplay of sleep in the circadian system’s phase responses to nighttime light exposure. The resulting data have been mixed, but generally support a modulatory role for sleep in circadian photic resetting (not an absolute requirement). *Drosophila* have been historically used to provide important insights in the sleep and circadian sciences. However, no experiments to date have evaluated how immediate sleep need or recent sleep history affects their pacemaker’s phase readjustments to light. We did so in the current study by (1) forcing separate groups of animals to stay awake for 1 or 4 h after they were shown a broadspectrum pulse (15 min during the first half of the night, 950 lux), or (2) placing them on a restricted sleep schedule for a week before light presentation without any subsequent sleep disruption. Forced sleep restriction, whether acute or chronic, did not alter the size of light-induced phase shifts. These data are consistent with observations made in other diurnal animals and raise the possibility, more broadly, that phototherapies applied during sleep—such as may be necessary during the winter months—may still be efficacious in individuals experiencing sleep-continuity problems such as insomnia.

## 1. Introduction

Mutual interactions between the circadian pacemaker and sleep homeostat guide choreographed transitions in sleep/wake behavior each day and shape the progression of internal sleep architecture across the subjective rest period [1,2]. Given the entwinement of these two systems, previous research has explored whether phase resetting of circadian rhythms by timed light exposure is influenced by immediate sleep need. Results have varied depending on the model tested. Sleep deprivation (induced by experimenter handling) in nocturnal wildtype mice and hamsters reduces the magnitude of light-induced phase shifts of the locomotor rhythm [3,4,5], but enhances these shifts in day-active rodents such as grass rats (Arvicanthis ansorgei) [6]. In humans, restricting sleep opportunity by housing people under short winter-like photoperiods attenuates melatonin phase responses to bright light administration [7,8]; however, early night sleep disruption caused by pharmacological arousal with caffeine has no effect [9]. *Drosophila* have long been used as platforms for reaching fundamental understandings of both circadian timekeeping and homeostatic sleep regulation [10,11]. The effect of sleep pressure on photic resetting of fly wake–sleep cycles has never been quantified despite the attention this question has increasingly received over the past two decades in people and other mammals. We attempted to fill this void with the current study, asking whether the magnitude of light-induced phase shifts in *Drosophila* was predicated on the animals sleeping within 4 h after presentation of a light stimulus or having a well-rested, undisturbed sleep history.

## 2. Results

**Figure 1:**
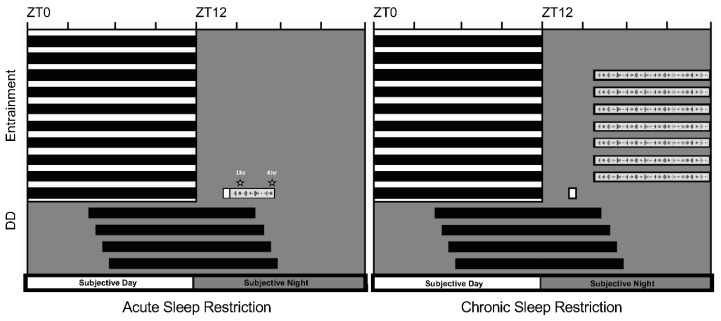
Overview of sleep restriction protocols. (**Left panel**) Independent groups of flies were sleep-restricted via mechanical perturbation for 1 or 4 h immediately following exposure to a 15-min broadspectrum light pulse (950 lux) at ZT14, 2 h after lights-off (19.00 MST) on the last day of entrainment to a 12:12 LD cycle. Phase shifts were quantified after free-running in constant darkness (DD) for 3–4 days. (**Right panel**) A separate group of animals was pulsed at ZT14 (15 min, 950 lux) after undergoing a chronic sleep restriction protocol (8 out of 12 h each night) for 7 consecutive nights. Once pulsed, the animals remained undisturbed for the rest of the experiment.

**Figure 2:**
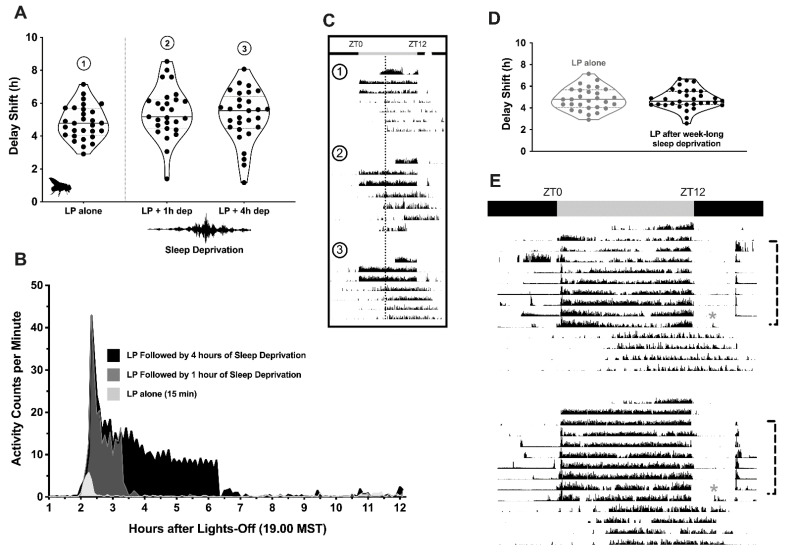
Acute and chronic sleep restriction: Lack of effect on circadian phase resetting. (**A**) Separate groups of flies received a 15 min pulse of uninterrupted broad-spectrum light (950 lux) at ZT14, 2 h after lights-out in a 12:12 LD cycle. One cohort remained undisturbed after the light pulse (LP alone; Condition 1, *n* = 29), while two other groups underwent sleep restriction via mechanical perturbation for either one (LP + 1 h dep; Condition 2, *n* = 27) or four (LP + 4 h dep; Condition 3, *n* = 29) hours. Violin plots show the magnitude of the delay shift in each animal’s locomotor activity rhythm after each treatment (1 point = 1 fly). The overall distribution of the data points is reflected by changes in width that occur along each plot. The median value and quartiles of each sample are marked with a thick solid line and thinner dotted lines, respectively. (**B**) Time series graph of each group’s activity counts (beam crosses min^−1^) across the remaining portion of the subjective night in which the light exposure and sleep restriction occurred. Flies who were exposed to light, but not mechanical perturbation, saw a slight uptick in their activity that declined within 10 min after the exposure had ended (marked in light gray). Those who were sleep-restricted for 1 or 4 h (marked with dark-gray and black data series, respectively) saw significant increases in behavioral activity within the first hour of the mechanical perturbation being applied; thereafter, activity plateaued to a steady rate during the rest of the sleep-restriction period, and then disappeared within 2 h of the perturbation being discontinued. (**C**) Representative actograms taken from flies receiving only light (Condition 1, Row 1), light and 1 h of sleep deprivation (Condition 2, Row 2) or light and 4 h of sleep deprivation (Condition 3, Row 3). Black bars indicate 30 s epochs where the animals registered breaks in the activity-tracking beams, smoothed over each minute of recording. Data are vertically aligned, such that one 24 h day of movement is shown per line, with successive days appearing one below the other. The 12 h portion of the LD schedule where the lights are on is centered (07.00–19.00 h, ZT0–12). An open white circle marks the timing of the light pulse. Example actograms from each protocol are organized in temporally aligned columns to help visualize shifts in locomotor rhythms with the aid of a dotted line. (**D**) Scatter violin plots summarizing the phase shifts in activity that were observed in flies without a history of sleep restriction (light gray) and those undergoing 7 consecutive nights of 8 h/night restriction (black). (**E**) Representative actograms taken from animals submitted to the chronic sleep-restriction protocol. Gray- and black-bar overhangs delineate the timing of the previous LD schedule. Asterisks mark delivery of the light pulse, while the brackets situated to the right of each actogram indicate the nights where mechanical perturbation was applied (between 23.00 h and 07.00 h, MST).

Sleep deprivation for 1 or 4 h pursuant to light presentation in the early part of the night at ZT14 (2 h after lights-off) did not impact circadian resetting of fly locomotor rhythms (protocol illustration in Figure 1, data shown in Figure 2A).

Animals were demonstrably active during the period in which mechanical perturbation was applied (and remained so for approximately 2 h after the gentle shaking had stopped; Figure 2B); however, the average delay shift observed in rested versus unrested flies was not statistically different (mean ± SEM; light pulse alone, 4.83 ± 0.19 h; light pulse and 1 h sleep deprivation, 5.43 ± 0.30 h; light pulse and 4 h sleep deprivation, 5.21 ± 0.29 h; *F*_3,113_ = 1.595, *p* = 0.1946; representative actograms provided in Figure 2C). Results were similar in *Drosophila* that had been partially sleep-restricted for a week before light exposure (Figure 2D,E; Dunnett’s multiple comparisons test, *p* > 0.99). The magnitude of delay shifts recorded in animals enduring several nights of limited sleep opportunity (4.80 ± 0.17 h) were not distinguishable from either those experiencing short-term sleep restriction or those with a normal sleep history before and after the light pulse.

## 3. Discussion

Our data suggest that *Drosophila* undergoing short or long-term sleep restriction have comparable circadian responses to light exposure in the first half of the night as well-rested animals, consistent with a prior report in a diurnal rodent that light-induced circadian phase shifting is not impaired when sleep pressure is elevated; in fact, it may even be potentiated with sleep pressure in animals such as grass rats, which were used in the aforementioned study [6]. These data may speak to differences in how sleep factors into the resetting of circadian timekeeping in diurnal versus nocturnal species. While previous studies in humans may argue against this possibility [7,8], it is important to note that in those studies, attenuation of circadian responses was achieved through the aggregate effect of partial sleep deprivation as well as photoperiodic history (with increased exposure to ambient evening light during the short nights) [8]; see author discussion. Whatever the dichotomy, a larger point generally emerges when comparing the phase-shifts to light made by diurnal and nocturnal animals who have slept and those who have not: whether in a fly, mouse, hamster, rat or human, an increase in sleep pressure caused by sleep deprivation modulates the size of the phase-shift by only 25–30% [3,4,5,6,7,8,9]. In so many words, light-mediated repositioning of the circadian pacemaker’s phase—while under some influence of the sleep homeostat—occurs largely independently of sleep homeostasis, a proposition commensurate with a voluminous literature documenting several functional dissociations between the circadian pacemaker and sleep homeostat e.g., [12,13,14,15,16,17]. With that said, it is important to note that there might be knock-on health and lifestyle effects associated with sleep deprivation that do influence the pacemaker’s phase-shifting responses to nighttime light exposure, potentially complicating any relationship that might be observed between sleep pressure and circadian photic resetting under real-world conditions or during short-term human in-lab experiments.

Ultimately, the significance of the current findings (as contextualized in the literature) is promising from the standpoint of using precision light emission devices to rehabilitate the circadian system during sleep [18,19]. Many neurological and psychiatric conditions are accompanied by interlocking episodes of circadian dysrhythmia and sleep loss, where preexisting sleep difficulties have the potential to interfere with the clinical goals of phototherapy. Our data suggest that sleep disturbances will not eliminate (or heavily compromise) the brain’s ability to respond to tailored schedules of light exposure that will orient its circadian function. Such interventions—unfettered by considerations of sleep quality and length—may be particularly beneficial for coordinating biological processes across the brain and body, thereby maximizing physical and mental health outcomes for individuals battling chronic disease.

## 4. Materials and Methods

High-resolution activity monitors were used to track and visualize circadian variations of behavioral activity in diurnal flies for each experimental protocol [20]. *Drosophila ananassae* were expanded from an isofemale line maintained at the National Drosophila Species Stock Center (NDSSC) at Cornell University (stock # 14024-0371.16). Through the embryonic and larval stages, the animals were maintained in DigiTherm^®^ incubators at 25 °C (Tritech Research, Inc., Los Angeles, CA, USA) and entrained to a 12:12 light-dark (LD) cycle (broad-spectrum light source: 4-watt cold-cathode fluorescent light tube with step-up inverter, illuminance at rack level = average of 887.7 lux; lights-on at 07.00 h, MST). For studies on sleep deprivation and phase-shifting, female flies were selected as late-stage “pharate-adult” pupae, moved onto fresh food, and housed in groups of 5 to 6. Three days post-eclosion, individual animals were placed without anesthesia into Pyrex glass chambers (5 mm outside diameter, 80 mm long) containing a plug of corn flour nutritional yeast–agar medium on one end (0.8% agar, 3.5% sucrose, 1.7% glucose, 6% fine-grained masa, 1% yeast) and a cotton fitting on the other. They were then loaded into MB5 monitors, where their motion was detected and counted with 17 independent infrared beams stationed across the 80 mm length of the housing tube (i.e., providing a spatial resolution of approximately 4 mm; TriKinetics, Waltham, MA, USA). Movement information from the beams was transmitted over modem/USB to a computer acquisition software every 30 s. MB5 units were situated in a climate-controlled vivarium (DR-36VL, Percival Scientific Inc., Perry, IA, USA) comparable to the ones used in colony management and under comparable ambient lighting conditions (950 lux, white fluorescent lighting, 4100K; Philips, F32T8/TL841, 800-series 32 W; lights-on at 07.00 h MST).

An Aschoff Type II paradigm was used to quantify the effects of light exposure (with and without sleep deprivation) on phase resetting of locomotor activity rhythms [21]. Flies continued entrainment to the 12:12 LD schedule under which they were reared for 3 more days. After lights-off on the last day of the schedule, separate cohorts were administered a single 15 min pulse at ZT14 (21.00 h, MST) by software-controlled activation of the house luminaire (950 lux, white fluorescent light; Percival IntellusUltra Controller). Postpulse, a control group of flies was left undisturbed for the remainder of the night phase. For two other independent sets of animals, light exposure was immediately followed by an acute period of mechanical sleep deprivation for either 1 or 4 h using a cushioned vortexer (1000 RPM; VWR International, Radnor, PA, USA) customized with a mounting plate for the activity monitors (Trikinetics model VMP-MB) (see protocol illustration in Figure 1). The vortexer was programmed to stir the animals awake at random 3 s intervals every 15 s across the two sleep deprivation periods with a computer-interfaced controller operating under the direction of Trikinetics DAMSystem3 software [22,23]. A fourth and final cohort of flies was shown light at ZT14 after being subjected to a week-long course of sleep restriction; under this protocol, the animals were given an opportunity to rest for the first 4 h of the night phase (12:12 LD cycle, lights-off at 19.00 h, MST), but kept awake for the remaining 8 h between 23.00 h and 07.00 h each night for 7 consecutive nights (see Figure 1, right panel). On the 8th night, they were given the 15 min light pulse without further sleep disruption.

On the heels of the various treatments, flies were left to free-run in DD for 3–4 days. Actogram plots reflecting daily locomotor activity were created by binning raw 30 sec time series data of individual animals. Phase shifts of behavior were calculated by determining the horizontal distance between regression lines fitted through software-called activity onsets 2 days prior and 2–4 days after light administration (ClockLab Analysis, Actimetrics, Wilmette, IL, USA) [24,25]. Changes in phase-shift magnitude resulting from the various sleep deprivation conditions were evaluated by one-way ANOVA with Dunnett’s post hoc correction. Significance was set at *p* = 0.05. In total, 117 animals were tested.

## Data Availability

The data that support the findings of this study are available from the corresponding author, F-X.F., upon reasonable request.
